# Functional Dyspepsia in Review: Pathophysiology and Challenges in the Diagnosis and Management due to Coexisting Gastroesophageal Reflux Disease and Irritable Bowel Syndrome

**DOI:** 10.1155/2013/351086

**Published:** 2013-05-16

**Authors:** Shadi S. Yarandi, Jennifer Christie

**Affiliations:** Division of Digestive Diseases, Emory University School of Medicine, Atlanta, GA 30322, USA

## Abstract

Functional dyspepsia is a common disorder which imposes significant diagnostic and treatment challenges for patients and physicians. The most recent update of the diagnostic criteria subdivides functional dyspepsia into two subcategories based on the main symptom of epigastric pain or postmeal fullness. As we discuss in this review, several studies have shown significant overlap in symptoms and pathophysiology between functional dyspepsia, irritable bowel syndrome, and the spectrum of reflux disorders. This overlap in symptoms can be informative in helping us to understand the underlying pathophysiology, diagnostic approaches, and treatment strategies. The addition of diagnostic testing such as pH impedance manometry of the distal esophagus to the current common diagnostic tests might be helpful in distinguishing between functional dyspepsia and reflux disease. Importantly, various treatment modalities may be more effective than others if the main symptom is burning rather than pain or postmeal fullness rather than early satiation.

## 1. How Is Functional Dyspepsia Defined?

Functional dyspepsia (FD) is one of the most common disorders of the upper gastrointestinal tract. According to Rome III criteria, it is defined as the presence of postprandial fullness, early satiation, epigastric pain, or burning in the absence of organic disease to explain the patients' symptoms. The Rome III criteria further subdivide FD into postprandial distress syndrome (PDS) and epigastric pain syndrome (EPS). The cardinal features of PDS are early satiation and sense of epigastric heaviness after a meal while the main feature of EPS is pain or a burning sensation in the epigastric area.

The definition of functional dyspepsia (FD) has been challenging and despite multiple changes in the definition of FD, the challenges are not entirely addressed. Additionally, the diagnosis as well as the management of this condition remains a clinical dilemma for physicians. One important challenge in defining and hence managing FD is the presence of coexisting reflux disease and irritable bowel syndrome (IBS) in many patients. Efforts have been made to separate these conditions by emphasizing features such as location of the symptoms, postprandial changes, or relieving factors. However, separating these features does not comprehensively differentiate between these conditions. For example, recent studies have shown that 37% of patients complaining of dyspeptic symptoms who fit in the category of EPS also have esophageal acid reflux proven by pH monitoring despite normal endoscopy [1].

Also, in patients diagnosed with functional heartburn, there is a high prevalence of dyspeptic complaints such as epigastric burning [2]. Additionally, there is a significant overlap between the diagnosis of EPS based on patient questionnaires and NERD based on pH monitoring [3]. This data suggests that perhaps using clinical features such as location of the burning sensation to differentiate between reflux disease and dyspepsia is not so reliable, and using more tests such as pH monitoring may be warranted.

In the first version of the Rome criteria, Rome I, FD was defined as the presence of pain or discomfort centered in the upper abdomen, in the absence of organic disease. Two subgroups were further defined as ulcer-like and dysmotility-like symptoms. Irritable bowel syndrome (IBS) symptoms and some reflux-associated symptoms such as heartburn were not excluded from the definition. According to the Rome II criteria, the definition of FD did not change significantly, but heartburn and IBS symptoms were excluded [4]. In both Rome I and Rome II, ambiguity of the term “discomfort” was a challenge to clinicians and researchers. Discomfort could include heaviness, early satiety, nausea, belching, or any other nonspecific terms used by patients to describe their symptoms. In Rome III, efforts have been made to avoid this ambiguity and use better-defined terms to describe dyspepsia such as pain, burning sensation, postprandial fullness, and early satiety [5] (Table 1).

## 2. What Do We Know about the Pathophysiology of FD?

Several factors have been studied that are thought to contribute to the pathogenesis of FD. In this section, we will briefly review some of these factors which include infectious factors, postinfectious changes in the gut, abnormal gastric motility, visceral hypersensitivity, and psychosocial and genetic factors (Table 2). Despite years of research, evidence regarding the role of these factors remains controversial, and it has been difficult to prove a causal relationship between any of these factors and the symptoms of FD.

### 2.1. Infectious and Postinfectious Etiology

Infectious causes such as *Helicobacter pylori* (*H. pylori*) have been linked to FD. Saito et al. studied the effect of long-term *H. pylori* infection on gastric emptying. They showed that prolonged *H. pylori* infection causes increased thickness of muscular layer of the stomach resulting in accelerated gastric emptying. To investigate the underlying pathogenesis of this process, they analyzed the miRNA expression profile in the stomach of the infected mice. MicroRNAs (miRNAs) are small noncoding RNAs that function as endogenous silencers of target genes, thus playing a critical role in cell proliferation, apoptosis, and differentiation. Saito and colleagues found a significant downregulation of miR-1 and miR-133 in muscular layer as a result of prolonged *H. pylori* infection. Additionally, both miR-1 and miR-133 are highly expressed in differentiated muscle tissues and control muscle differentiation as well as proliferation. One may hypothesize that downregulation of miRNA may cause hyperplasia of muscular layer, leading to accelerated gastric emptying and the development of dyspeptic symptoms [15]. 

However, translation of basic research findings regarding the role of *H. pylori* infection in FD into clinical practice has not been completely successful. Double-blinded, randomized control trials investigating the effect of *H. pylori* eradication in human have produced controversial results [20, 21]. While Miwa et al. reported no benefit of *H. pylori* infection eradication for FD symptoms, another study in Asian population did show improvement in symptoms. It seems that the effect of *H. pylori* eradication is minimal, with the number needed to treat being 15 to achieve a small effect size [22]. In addition, histological studies failed to show any correlation between the severity of inflammation and presence of dyspepsia.

Other postinfectious causes have been investigated in relation to the onset of FD. An increased prevalence of dyspeptic symptoms has been reported in the general population after an outbreak of bacterial gastroenteritis especially secondary to a *Salmonella* or viral infection presenting with nausea and vomiting [16]. There is evidence of increased infiltration of eosinophils, macrophages, and intraepithelial lymphocytes in patients with postinfectious dyspepsia [18]. Recently, it has been shown that increased cytokine levels and a specific type of T-lymphocyte homing are associated with higher intensity of pain, cramps, nausea, and vomiting but not fullness or satiety in patients with postinfectious dyspepsia [23]. Most of this evidence remains sporadic, and the clinical significance of postinfectious inflammatory changes observed in histological studies needs to be clarified by further studies.

### 2.2. Gastric Motility

One of the most frequently studied mechanisms implicated in the development of FD is abnormal gastric motility. Studies have reported two types of gastric motility abnormality as the underlying mechanisms for FD. These include abnormal accommodation of the gastric fundus and abnormal gastric emptying. Tack et al. showed that immediate accommodation of gastric fundus to food is impaired in patients who complain of early satiation [6]. Additionally, more than two-thirds of patients with FD have an abnormal electrogastrography on electrophysiological studies reflected by a reduction in gastric slow waves in both fasting and postprandial states [24]. 

However, postprandial pain and heaviness have been attributed to both delayed and accelerated gastric emptying. Earlier studies reported that delayed gastric emptying is seen in patients with heavy feeling after food [7]. Interestingly, delayed gastric emptying in patients with FD has been attributed to the effect of ghrelin, a motilin-related GI peptide, but this has not been confirmed with further studies [25–27]. Recently, the role of delayed gastric emptying in the pathogenesis of postprandial symptoms has been challenged by a study from Kusano et al. [8]. In 8 patients with PDS variant of FD, they found that accelerated gastric emptying rather than delayed gastric emptying is associated with heavy feeling after meals. They reported this phenomenon to be worse after liquid fatty meal and attributed this effect to the reflexive increase in the secretion of cholecystokinin (CCK). The evidence reviewed above suggests that the underlying mechanisms for postprandial pain and early satiation are different, and our understanding of the role of abnormal gastric motility in the pathogenesis of these symptoms is far from complete.

### 2.3. Visceral Hypersensitivity and Altered Sensation

Visceral hypersensitivity is also associated with the development of FD. Specifically, gastric hypersensitivity has been shown to be due to several different factors. Hypersensitivity has been shown to be associated with gastric distension, gastric acid, and bile. Studies have shown that patients with FD, especially those who complain of postprandial epigastric pain, experience pain at a lower level of inflation of a barometer in the stomach [9], suggesting that an increased sensitivity to mechanical stretch may be the source of the epigastric discomfort.

Furthermore, it has been shown that the level of acid secretion in FD patients is not elevated [28], but there is hypersensitivity of the duodenal mucosa to normal gastric acid [10]. Cao et al. have also shown that chemosensitivity to capsaicin is increased in patients with FD. In this study, a lower amount of capsaicin was required to induce pain in patients with FD compared to healthy subjects [29]. However, Hammer et al. failed to show any correlation between hypersensitivity to capsaicin and any specific symptom or severity of symptoms in FD patients [30].

Additionally, hypersensitivity has been suggested to be perceived at a central sensory level, with glutamate as the potential neurotransmitter involved. This theory suggests that increased presynaptic release of glutamate in the central sensory areas facilitates transmission of visceral sensory signals, leading to an amplified response to nonpainful stimuli and perception of pain. In addition, central hypersensitivity can potentially lead to activation of previously silent visceral nociceptors through recruiting more spinal neurons to the pain pathway [31]. Furthermore, functional imaging studies have been done in patients with FD and have displayed abnormal regional brain activity in these patients suggesting a central nervous system effect [32, 33].

### 2.4. Genetic Factors

Evidence suggesting the role of genetic factors in FD originates from studies that have shown that patients with a positive family history of FD are more likely to have symptoms of dyspepsia [34]. Studies have suggested that polymorphism of G-protein b3 (GNB3) subunit gene (C825T) is more prevalent in patients with FD. G-proteins function as membrane receptors, and their dysfunction interferes with intracellular signal transduction. The GNB3 825T allele is associated with enhanced G-protein activation that might cause dysfunction of adrenoreceptors mediating visceral sensation and motor function of GI tract. However, it is unclear which subtype of FD is associated with this genetic polymorphism. While some studies suggested a link between polymorphism of C825T and EPS subtype of FD [11, 35], others have reported a link between this genetic polymorphism and PDS subtype of FD [36]. Furthermore, a recent study has reported increased prevalence of the polymorphisms in this gene in patients with concurrence of FD and IBS [37].

Serotonin transporter protein (SERT) is another protein that has been suggested to be involved in the pathogenesis of FD. This protein is coded by 17q11 and is involved in the reuptake of serotonin at the synaptic cleft in gastrointestinal tract. Polymorphism in the gene coding this protein has been linked to FD, specifically PDS subtype [12], although results have been controversial and other studies failed to confirm the link [35, 36]. 

The migration inhibitory factor (MIF) gene polymorphisms are reported to be associated with increased risk for development of EPS symptoms in Japanese population. Additionally, in *H. pylori*-infected patients, polymorphism of IL-17F gene is associated with higher prevalence of EPS [13]. Both IL-17F and MIF have important regulatory roles in immune and inflammatory response to pathogens such as *H. pylori*, and modification of their structure or functions can affect GI immune response to infection.

### 2.5. Psychosocial Factors

Psychosocial factors are well-known contributors in pathogenesis of FD. There is a higher prevalence of psychological symptoms in patients complaining of dyspepsia. A large-scale epidemiologic study showed that anxiety is more common in patients with the diagnosis of FD; however, they did not have a higher depression score [38]. Another recent large-scale cohort study of 1175 patients showed that among people free of a FD at baseline, higher levels of anxiety but not depression at baseline were a significant independent predictor of developing new onset FD 12 years later [39].

In a high percentage of FD patients, symptoms can be aggravated by cognitive factors. For example, one study showed that low fat diet can exacerbate the symptoms if patients perceive the consumed food as high fat [40]. It has also been reported that mental stress is associated with aggravation of postprandial symptoms that might be related to sympathetic hyperactivation and elevated levels of corticotropin releasing hormone (CRH) which has been shown to delay gastric emptying [19].

A recent study examined the correlation between subtypes of FD, psychiatric abnormalities, and personality traits. PDS was independently associated with somatization (correlation coefficient: 0.28, *P* = 0.034), depression (correlation coefficient: 0.27, *P* = 0.028), and phobia (correlation coefficient: 0.24, *P* = 0.044) while EPS was not significantly correlated with any psychiatric abnormality [41]. Somatization has also been linked to the prevalence and severity of FD symptoms in other studies as well [42, 43].

### 2.6. Other Factors

Lastly, several other factors have been associated with symptoms of dyspepsia including environmental, dietary factors, and lifestyle. There are also sporadic reports of role of the melatonin and neural autoantibodies in the pathogenesis of dyspeptic symptoms but their relevance remains to be proven [44, 45]. It is likely that the interaction of more than one factor produces symptoms of dyspepsia. 

Overall, the pathophysiology of FD is complex with many factors involved. Some of them are modifiable like psychosomatic disorders or increased gastric acid secretion; some of them are not such as genetic polymorphisms. Nevertheless, no single factor has shown convincing causation of dyspepsia. Therefore, further studies are needed to focus on the interaction of several factors in the pathogenesis of FD.

## 3. What Is the Evidence for Coexistence of FD, IBS, and GERD?

Before Rome III criteria were developed, several studies have reported the coexistence of FD, GERD, and IBS using the Rome II criteria. For example, one study reported a markedly high rate of coexisting upper and lower GI symptoms in patients with IBS (75% concurrence rate), especially in patients with constipation as their predominant symptom [46]. Another large study in a tertiary setting showed a significant concurrence of GERD and IBS and reported high prevalence of dyspepsia (23%) along with extraintestinal pain such as headache (27%) and low back pain (16%) in the group with coexisting symptoms [47].

After Rome III was published, several reports on the coexistence of IBS, GERD, and FD based on the new definition were published. The focus of these studies was mostly to validate the presence of overlapping symptoms after the change in the definition and also to investigate the effect of coexistence of these conditions on health-related quality of life.

Among the studies that examined overlap of FD and IBS, a large Chinese population study by Wang et al. reported results of a survey analysis of patients in an outpatient gastroenterology clinic. Among 3014 patients who participated in the study, 608 fulfilled the criteria for FD and 480 for IBS. About 25% of patients with FD also fulfilled the criteria for IBS while 31.5% with IBS also were diagnosed with FD. The prevalence of IBS in the PDS subtype was significantly higher than EPS, although most of patients with IBS and FD had a combination of PDS and EPS phenotypes [48]. This data represents a strong overlap of FD and IBS. This finding has been confirmed in other ethnic groups as well. For example, Van Oudenhove et al. showed that FD has a significant concurrence rate with IBS (56%) and also chronic fatigue syndrome (40%) in a Dutch population [49]. One limitation of both of these studies was that their patients were recruited from a referral gastroenterology clinic that might lead to overestimation of the overlap of FD and IBS in the general population.

However, studies done in the primary care setting have produced similar results. Kaji et al. reported that among 3125 patients in the primary care setting, the prevalence of FD and IBS was 10% and 14.4%, respectively. Among these patients, 106 patients showed overlap between symptoms of FD and IBS. Furthermore, these patients showed significantly lower scores on health-related quality of care questionnaires [50].

Other studies have illustrated the coexistence of FD and the spectrum of reflux disorders. For example, Ohara et al. analyzed 1115 patients who presented to primary care clinic with complaints of heartburn, epigastric pain, or burning and reported a 10% overlap of FD and GERD based on the results of a symptom questionnaire [3]. However, 91% of their participants also had findings of reflux esophagitis on endoscopy. However, they did not report the prevalence of concurrence between FD and NERD. 

However, Noh et al. separately reported the prevalence of FD in patients with GERD and NERD. They studied 2388 patients in primary care setting and reported that FD is found in patients with GERD as well as NERD. However, the rate of overlapping symptoms was significantly higher in patients with NERD than patients with reflux esophagitis: 74.3% and 10.5%, respectively. They additionally examined the prevalence of FD subtypes and showed that while in patients with NERD, EPS subtype was more prevalent (68.9% EPS versus 48.6% PDS), PDS subtype was more prevalent in patients with reflux esophagitis (5.2% EPS versus 7.3% PDS) [51]. This important finding was also confirmed by Savarino et al. who showed that among 200 patients with NERD and functional heartburn, the prevalence of dyspeptic symptoms including epigastric pain and burning was high (23–61%) [2]. This evidence shows that normal endoscopy findings in patients with dyspepsia does not rule out the concurrent presence of reflux due to the high prevalence of coexisting NERD and FD. In a particularly interesting study, Xiao et al. analyzed 186 patients who were diagnosed with FD based on Rome III and performed pH monitoring. They reported the presence of pathological acid reflux in 32% of patients with FD and normal endoscopy [1].

Idiopathic gastroparesis is another condition that might be difficult to differentiate from FD. About 40% of patients with FD have delayed gastric emptying, and patients with idiopathic gastroparesis can present with symptoms similar to FD. National Institute of Diabetes and Digestive and Kidney Diseases Gastroparesis Clinical Research Consortium has recently reported that the rate of overlap between symptoms of idiopathic gastroparesis and FD is about 87% [52]. Based on this result, some authors have questioned whether or not these are separate entities and have suggested that gastric emptying studies should be considered in the initial workup, particularly if the symptoms are mostly postprandial pain, nausea, and vomiting [53]. However, gastric emptying measurements have shown poor correlation with the severity of the symptoms and need to be validated for a more accurate differentiation between these conditions [52, 54].

## 4. What Are the Diagnostic and Management Implications of Coexisting FD, GERD, and IBS?

There are several conclusions that can be derived from the evidence demonstrating a high prevalence of coexisting reflux, IBS, and FD. Importantly, identifying the cardinal symptom or chief complaint of the patient is essential to deciding the appropriate diagnostic tests and selecting the appropriate method of treatment. For example, in patients who are diagnosed with the EPS subtype of FD, the evidence suggests that if the main symptom is burning sensation at the epigastrium, there is a high probability of a coexisting reflux disorder. Therefore, treatment with acid suppression should be considered the first line of therapy. Alternatively, treatment with acid suppressing agents might not be as effective in EPS patients with epigastric pain. Furthermore, acid suppressing therapy is not the first line of treatment for patients with PDS subtype for whom prokinetics may be more appropriate.

Conversely, the evidence reviewed here highlights the fact that the symptoms alone are not exclusively reliable in making the diagnosis, and appropriate tests should be used. For example, the location of the burning sensation (sub-sternal versus epigastric) which has been previously used to separate FD from reflux disorder has limited value since many people with reflux disorder have a burning sensation at the epigastrium, and many with substernal burning do not have any evidence of reflux on pH monitoring. Differentiating these conditions has significant effect on management since patients with functional heartburn or FD without evidence of acid reflux might benefit from other therapy such as tricyclic antidepressants [55] or psychological management. Furthermore, it has been shown that adding impedance pH testing to Rome III criteria adds diagnostic value to the diagnosis of reflux versus FD and should be considered as part of initial evaluation [56].

Coexistence of symptoms of FD and IBS is well documented in the literature. This coexistence is more pronounced in constipation dominant variant of IBS in which patients with FD may experience a feeling of fullness and early satiation. However, in one longitudinal study of patients with IBS and FD, 40% of patients converted their clinical presentation from IBS to FD or vice versa over a 12-year follow-up period [57]. In this study, the definition of these conditions was mutually exclusive, so patients could not belong to more than one category. However, it is possible that both conditions were present simultaneously with one being more prominent. 

Furthermore, FD and IBS share common pathophysiology and symptoms. In both conditions, there is increased sensitivity to gut or stomach distention and a decreased threshold for pain. Delayed gut transit time as well as delayed gastric emptying is another common feature of these conditions [58]. Common pathophysiologic features and common clinical symptoms might suggest that FD and IBS are not separate disorders but are parts of one single spectrum of a disease that can be called “irritable gut” [59]. 

An important question to ask is as follows: does considering these entities as separate or part of the same process change the way clinicians manage these diseases? From what we have learned, it seems that at least for the PDS subtype of FD and constipation predominant IBS, the management strategy can be very similar in regards to dietary and lifestyle modification as well as the use of prokinetic medications.

**Figure 1:**
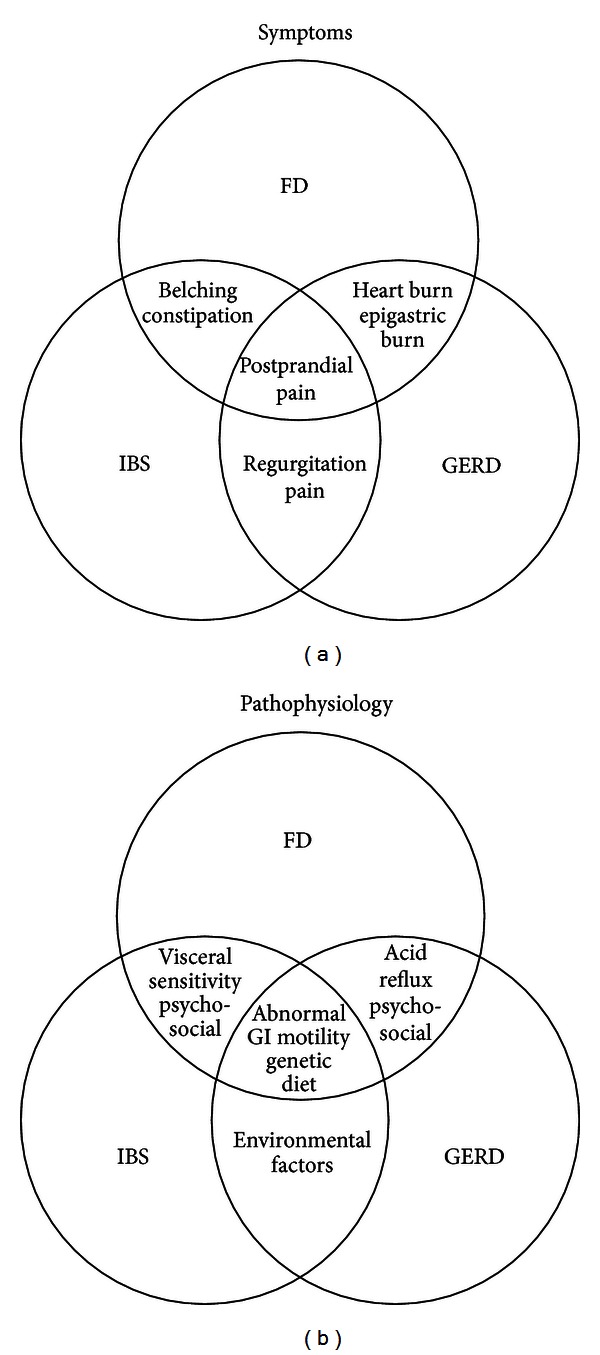
Overlapping symptoms and pathophysiology of FD, GERD, and IBS. (a) Overlap between symptoms of FD, IBS, and GERD. Postprandial pain can be present in all three conditions. Heartburn can be seen in patients with FD in the absence of acid reflux while epigastric pain can be caused by reflux disorder. (b) Overlap between pathophysiology of FD, IBS, and GERD. Genetic factors are involved, and abnormal GI motility is the common mechanism in all three conditions.

In summary, the accurate diagnosis of functional disorders and separating them from concurrent disorders such as reflux disorder remains a significant challenge for physicians. Most of the symptoms used to differentiate between FD, IBS, and GERD or to define subtypes of FD such as postprandial pain or burning sensation can be seen in any of these conditions. Current diagnostic models based on clinical presentation fail to differentiate between variants of FD, GERD/NERD, and IBS with high specificity and sensitivity, leaving the field open for further study to capture the complexity of the interaction between symptoms and underlying pathophysiology (Figure 1). Further studies are required to elucidate the role of incorporating further diagnostic studies such as impedance pH monitoring into current diagnostic algorithms.

Our understanding of the pathogenesis of functional bowel disorders, although still far from complete, has improved significantly over the recent years. Increasing data regarding the involvement of genetic factors has provided us with new insights into the pathogenesis of functional bowel disorders and with novel potential treatment targets. Increased recognition of the role of inflammatory cytokines and postinfectious changes has also enhanced our view of the pathogenesis.

Further research is required to advance these findings and to translate them into practical treatment methods. When selecting the method of treatment, physicians should consider and search for overlapping symptoms as well as concurrent disorders, as this might change the preferred method of treatment.

## Figures and Tables

**Table 1:** Comparison of Rome II and Rome III.

	Rome II	Rome III
Duration of symptoms	12 weeks	12 weeks

Time period preceding diagnosis	12 months	6 months

Symptoms	Pain Discomfort	Bothersome postprandial fullness Early satiation Burning Pain

Location of symptoms	Centered in the upper abdomen	Epigastric

Need to rule out organic causes	Yes	Yes

Other exclusions	Not relieved by defecation Not associated with change in stool frequency or form	None

	Ulcer like Pain in the upper abdomen	Postprandial distress syndrome Bothersome postprandial fullness after meals Early satiation before finishing a regular meal
Subtypes (feature/location of symptoms)	Dysmotility like An unpleasant nonpainful sensation centered in upper abdomen	Epigastric pain syndrome Pain or burning at epigastrium Pain of at least moderate severity Pain is intermittent, not relieved by defecation Pain not caused by gallbladder or sphincter of Oddis dysfunction pain
	Unspecified Symptoms do not fit in the above categories	

**Table 2:** Summary of proposed mechanisms for functional dyspepsia.

Pathogenesis	Proposed mechanism
Abnormal gastrointestinal motility	(i) Abnormal accommodation of gastric fundus [6] (ii) Delayed gastric emptying [7] (iii) Rapid gastric emptying [8]

Visceral hypersensitivity	(i) Increased sensitivity to mechanical stimulation (gastric dilation) [9] (ii) Increased sensitivity to chemical stimulation (gastric acid or bile) [10]

Genetic factors	(i) Increased risk of FD in patients with polymorphism of G-protein b3 (GNB3) gene [11] (ii) Increased risk of PDS subtype of FD with polymorphism of serotonin transporter protein (SERT) gene [12] (iii) Increased risk of EPS subtype of FD with polymorphism of migration inhibitory factor (MIF) gene [13] (iv) Increased risk of EPS subtype of FD with polymorphism of regulated upon activation of normal T cells expressed and secreted (RANTES) gene [14]

*H. pylori* infection	Downregulation of miR-1 and miR-133 caused by *H. pylori* infection [15]

Postinfectious causes	(i) Increased prevalence of dyspeptic symptoms after infectious gastritis [16] (ii) Increased expression of interleukin 1*β* [17] (iii) Increased infiltration of gastric mucosa with eosinophils, macrophages, and intraepithelial lymphocytes after infection [18]

Psychosocial factors	(i) Higher prevalence of psychological symptoms in patient with dyspepsia (ii) Stress-induced elevated levels of CRH and ACTH which can affect gastric emptying [19]

Other factors	(i) Environmental factors (ii) Dietary exposures
